# Special Issue “Molecular Advances in Cancer and Cell Metabolism”

**DOI:** 10.3390/ijms26051876

**Published:** 2025-02-21

**Authors:** Maria Mesuraca, Barbara Quaresima, Stefania Scicchitano, Maria Concetta Faniello

**Affiliations:** 1 Department of Experimental and Clinical Medicine, University Magna Græcia, 88100 Catanzaro, Italy; quaresi@unicz.it (B.Q.); scicchitano@unicz.it (S.S.); 2 Research Center of Biochemistry and Advanced Molecular Biology, Department of Experimental and Clinical Medicine, “Magna Graecia” University of Catanzaro, 88100 Catanzaro, Italy

Mammalian cells can obtain energy by taking up different macromolecules, depending on their availability in the external environment. These nutrient uptakes occur through transporter proteins or channels, as well as through endocytic processes [1]. Once inside the cells, metabolic processes are structured into complex networks that are regulated according to current physiological conditions and energy demands. In healthy tissues, oxidative phosphorylation meets metabolic needs to support vital functions. However, in hypoxic tumor environments, the rapid growth and dissemination of cancer cells lead to an adaptation process that requires alternative metabolic pathways for energy production [2,3,4]. In solid tumors, hypoxia-inducible factors (HIF) support cell survival in poor O_2_ conditions by increasing glucose transport and overexpression of glycolysis enzymes, regenerating NAD+ from NADH by upregulated activity of lactate dehydrogenase (LDH) and PDH-inhibitory kinase 1 (PDK1) enzymes thus decreasing mitochondrial respiration [1]. In addition, HIFs proteins active genes involved in cellular survival, proliferation and angiogenetic factors such as stromal-derived factor 1 (SDF1) and vascular endothelial growth factor (VEGF) supporting the tumor vascularization [5]. Certainly, neoplastic transformation makes use of multistep processes that include genetic and epigenetic changes that contribute to tumorigenesis; in recent times, numerous studies have highlighted the critical role of metabolic reprogramming and the ability to escape immunosurveillance as core of hallmarks of cancer, to create a favorable microenvironment contributing to cell growth and disease progression [6,7]. Cancer cells exhibit an increased uptake of glucose and amino acids, which limits the availability of essential molecules for immune cells. This altered metabolism produces oncometabolites from the tricarboxylic acid cycle, such as D-2-hydroxyglutarate (2-HG), which promotes aggressive phenotypes in breast cancer. Li et al. and Zhang et al. discuss these implications [7,8]. Additionally, Furth et al. reported that mutations in the isocitrate dehydrogenase 1 (IDH1) gene, commonly found in gliomas, lead to the production of 2-HG as a substitute for α-ketoglutarate. This study also demonstrated that mutant IDH1 contributes to the deregulation of histone acetylation through chromatin remodeling [9].

Lipid metabolism is essential for meeting the heightened energy demands of cancer cells. It not only generates energy, but also supplies structural components necessary for the formation of new membranes during rapid cell division. Additionally, lipid metabolism produces signaling molecules known as lipid mediators, which can promote tumor development through oncogenic pathways [10,11]. One key player in this process is ATP-citrate lyase (ACLY), which catalyzes the production of cytoplasmic acetyl-CoA exported from mitochondria via citrate. The overexpression of ACLY has been linked to various chronic diseases and cancers. Furthermore, ACLY’s activity in the nucleus plays a significant role in epigenetic regulation and histone acetylation [12]. A study conducted by Guo et al. demonstrated that in melanoma cells, ACLY enhances cancer growth and proliferation by activating acetyltransferase and promoting the transcription of the microphthalmia-associated transcription factor (MITF)–PPARγ coactivator-1α (PGC1α) axis [13].

Recent studies have increasingly demonstrated that noncoding RNAs (ncRNAs) are involved in metabolism reprogramming and epigenetic regulation [14]. For instance, Wang et al. found that miR-N5 can inhibit the acetylation of histone H3 at lysine 56 (H3K56) on the promoters of β-catenin and Epidermal Growth Factor Receptor (EGFR) genes. This inhibition reduces the migration and invasiveness associated with prostate cancer metastasis [15].

Tumor cells often exhibit an abnormal redox metabolism, which disrupts the balance between the generation of reactive oxygen species (ROS) and the intracellular antioxidant defense mechanisms. This imbalance can alter signaling pathways and affect genomic stability. In a recent study, Hagiwara et al. demonstrated that the oncogenic protein Mucin 1 (MUC1-C) leads to chromatin remodeling of the polybromin-associated BAF complex (PBAF) in prostate cancer, thereby supporting redox balance and the expression of NFE2-Related Factor 2 (NRF2) target genes. The authors emphasized that MUC1-C interacts with both the NRF2 and Polybromo 1(PBRM1) genes, enhancing the expression of the SLC7A11 and Glucose-6-Phosphate Dehydrogenase (G6PD) genes. This interaction promotes the activation of antioxidant genes, which are crucial for maintaining genomic stability [16]. Increased evidence suggests that NRF2 plays a central role in maintaining redox homeostasis. The activity of this gene is influenced by multiple factors and signaling pathways in solid tumors, making it a promising target for chemotherapy [17,18].

In tumor cells, molecular mechanisms and epigenetic alterations lead to a reprogramming that provides a selective growth advantage. This process contributes to both the initiation and progression of cancer, while also enabling the cells to evade the effects of drugs [2,19]. Understanding the cellular mechanisms behind these remodeling events can reveal potential vulnerabilities in neoplastic cells.

The aim of this Special Issue is to enhance our understanding of the complex mechanisms involved in tumor development and to identify potential metabolic targets that could increase drug sensitivity.
